# Identification of microsatellite loci in sea anemones *Aulactinia stella* and *Cribrinopsis albopunctata *(family Actiniidae)

**DOI:** 10.12688/f1000research.13724.1

**Published:** 2018-02-27

**Authors:** Ekaterina S. Bocharova, Alexey A. Sergeev, Aleksandr A. Volkov

**Affiliations:** 1 Russian Federal Research Institute of Fisheries and Oceanography, Moscow, 107140, Russian Federation

**Keywords:** cnidaria, sea anemones, microsatellites, primers, Aulactinia, Cribrinopsis

## Abstract

From the DNA libraries enriched by the repeat motifs (AAAC) _6_, (AATC) _6_, (ACAG) _6_, (ACCT) _6_, (ACTC) _6_, ACTG) _6_, (AAAT) _8_, (AACT) _8_, (AAGT) _8_, (AGAT) _8_, for two viviparous sea anemones *Aulactinia stella* and *Cribrinopsis albopunctata, *41 primer pairs were developed. These primer pairs resulted in the identification of 41 candidate microsatellite loci in either *A. stella* or *C. albopunctata*. Polymorphic loci were identified in both sea anemone species for 13 of the primer pairs and can be applicable for population genetics researches.

## Introduction

Sea anemones are known to live in clonal or partially clonal populations ( Bocharova, 2015; Bocharova 2016; Bocharova & Mugue, 2012). Data based on sequences of mitochondrial (12S rRNA, 16S rRNA and cytochrome oxidase III) and nuclear (18S rRNA and 28S rRNA) genes, which are successfully used in phylogenetic research, are not applicable to population genetics studies because of the high amount of monomorphic samples. Sometimes it is not evident that a population is clonal, for instance, in the parthenogenetic populations of *Aulactinia stella* (Verrill, 1864) in the White and the Barents Seas ( Bocharova & Mugue, 2012; Bocharova, 2015). Representatives of other species can combine sexual and asexual (clonal) reproduction in response to environmental changes ( Bocharova & Kozevich, 2011). For *Cribrinopsis albopunctata* Sanamyan et Sanamyan, 2006 there is no data about its asexual or parthenogenetic reproduction and populations of this species usually consist of males and females. Thus, these two species are characterized by different reproductive modes. The development of polymorphic microsatellite markers resulted in the design of 41 primer pairs, which were subsequently screened using DNA from both *A. stella* and *C. albopunctata* to assess primer utility in different species and populations of the same species.

## Methods

For this research, sea anemone specimens were collected in Avachinsky Bay of Kamchatka Peninsula at the depths of 11–18 meters and identified *in vivo*. The total DNA was extracted from the samples, which were preserved in 96% ethanol, using the Wizard SV Genomic DNA Purification System (Promega) with previous desiccation and grinding. Extracted genomic DNA was exposed to fragmentation by Covaris S-Series (Covaris, USA) resulting in average distribution of fragment lengths of about 150–200 bp, which were additionally estimated by capillary electrophoresis (Nanofor-05, Syntol, Russia) with non-denaturing polymer and intercalating dye. DNA libraries were prepared by using TruSeq DNA LT Sample Prep Kit (Illumina, USA). Then the libraries were enriched for the repeat motifs (AAAC) _6_, (AATC) _6_, (ACAG) _6_, (ACCT) _6_, (ACTC) _6_, ACTG) _6_, (AAAT) _8_, (AACT) _8_, (AAGT) _8_, (AGAT) _8_ and screened according to the protocol described in Glenn & Schable (2005). The fragments containing hybridization of biotinylated oligonucleotides with tandem microsatellite repeats was separated by magnetic Streptavidin M-280 Dynabeads (Dynal, Oslo, Norway). The enriched genomic DNA libraries were treated using Miseq Kit v2 for 300 cycles in the paired-end read mode (250 + 250 bp) for MiSeq next-generation sequencing (Illumina, USA).

Sequences obtained were analyzed for the repeat regions by NGS analysis tool Geneious 10.2.3, which also compared sequences to determine the existence of duplicates. This software was also used to create 41 primer pairs flanking the repeat regions of interest. Primers were named Act ## and numbered sequentially.

The total DNA from pedal disc tissues of five *A. stella* specimens and three *C. albopunctata* specimens was extracted by Wizard SV Genomic DNA Purification System (Promega, USA) following the manufacturer’s protocol. Extracted DNA was amplified using the newly created primers. An amount of 50 ng of the extracted DNA was amplified in 20 µL reactions with 1x SmarNGTaq Buffer (Dialat Ltd., Russia), 25 µM of each of four deoxyribonucleoside triphosphates, 2 mM MgCl _2_, 0.1 µM of each fluorescent labeled forward and unlabeled reverse primers, and 1 unit SmarNGTaq polymerase (Dialat Ltd., Russia). Amplification of all the microsatellite loci was performed by Touchdown PCR with the following conditions: 96°C for 3 minutes for initial denaturation, followed by 30 cycles at 96°C for 10s, 62°C for 30s (with a 0.2°C decrease in the second step of each cycle), 72°C for 10s; 10 cycles at 96°C for 10s, 56°C (with a 0.2°C increase in the second step of each cycle) for 30s, 72°C for 10s; 20 cycles at 96°C for 10s, 56°C for 30s, 72°C for 10s; 72°C for 10 minutes; ending with a 4°C soak.

One µL of PCR product was added to 24 µL of deionized formamide Hi-Di (Applied Biosystems, USA) and 1 µL of Liz-labeled ladder SD-450 (Syntol, Russia) and denatured at 95°C for 3 minutes. Products were visualized in 3500 Genetic Analyzer (Applied Biosystems, USA) using POP7 gel polymer.

## Validation

Analysis of the obtained chromatograms was performed by GenMapper Software (ThermoFisher Scientific, USA). Of the 41 primer pairs developed, 5 (12.2%) resulted in poor or no amplification in both *A. stella* and *C. albopunctata*. Almost half (56.1%) of the remaining loci successfully amplified was monomorphic in the two species. Finally, 13 primer pairs appeared to amplify polymorphic microsatellite loci at combined panels for the two species ( Table 1).

**Table 1.** Characterization of 13 polymorphic microsatellite loci in the pooled DNA of Aulactinia stella (5 individuals) and Cribrinopsis albopunctata (3 individuals).

	Primer sequence (5'–3')	PCR product length (bp)	Repeat motif	No. of alleles	Allele size range (bp)
Act007	F: TGCAACTAACCCAAGCACCT	199	(ACAG) _5_	2	192–196
	R: TCGTTGGCTGTCCTCTTGTC				
Act011	F: AACAACACATATAGGGTTACGTGTA	135	(AATC) _3_	3	137–165
	R: AATAGCCATAGAAGCTGGATGAATG				
Act020	F: CGATGCGACTAGGACCGTC	199	(AACT) _12_	4	203–215
	R: GCTTGGTGTTGGCATTGAGG				
Act021	F: TTACGATCTTCTGAGATTAAGCCTT	282	(AACT) _9_	5	268–284
	R: TAAAGGTCTACTGTTGTCTTATCCC				
Act028	F: TAAGCCTTTGTTCTACGATTTGTTC	299	(AACT) _24_	5	252–268
	R: GGTCTACTATTGTCTTATCCCTGAC				
Act061t	F: TGCAGTCATTCTACCCGCAA	289	(ATC) _20_	3	273–294
	R: ACCACAGGGCTAAACAAGACA				
Act173	F:TGCAACTAACCCAAGCACCT	196	(ACAG) _4_	2	197–201
	R: CTCGTTGGCTGTCCTCTTGT				
Act177	F: TTGAAATACTTGTAGAAATGGCACC	238	(AATC) _18_(AACC) _2_	5	176–244
	R: AACACATATAGGGTTACGTGTAGAC				
Act235	F: TGGACTTGCATCTTATAACCCTAGA	210	(AAAC) _6_(AAA)(AAAC) _3_	3	199–211
	R: GGTGTTCGACATTAACCTGCT				
Act238	F: TGTCCGTCTGATTGTCTGCC	121	(ACAG) _2_(ACAA)(ACAG) _2_(ACAA)	3	122–154
	R: GGAGTTCCTGAGTTTGCTGC				
Act249	F: ACGGTCATCAATTCGGCTCA	123	(AAAC) _5_	2	130–134
	R: TCCAATACAACGGTCACTCACT				
Act252	F: GTTGTCAGTTCCCGTCCAGT	133	(AAAC) _4_	2	131–135
	R: TTTGCCTTCCAACGAACAGC				
Act304	F: GGTGTTCGACATTAACCTGCT	200	(AAAC) _11_	4	192–204
	R: CGGTCCCTTATAACCCTAGAATCA				

## Data availability

The raw data is available:

- https://doi.org/10.5281/zenodo.1171106 ( Bocharova *et al.*, 2018a). The dataset contains three files in different formats (*.scv, *.geneious,*.fasta) with primer sequences of all 41 STR loci for *Aulactinia stella* and *Cribrinopsis albopunctata*.
- http://doi.org/10.5281/zenodo.1144120 ( Bocharova *et al.*, 2018b). The dataset includes *.fsa files (the Prism Genetic Analyzer 3500, Applied Biosystems) of pooled DNA PCR product of 13 STR polymorphic loci for *Aulactinia stella* and *Cribrinopsis albopunctata*.
