# Supplementary material for: Identification of microsatellite loci in sea anemones Aulactinia stella and Cribrinopsis albopunctata (family Actiniidae)
Source: F1000Res. 2018 Feb 27;7:232. [Version 1] doi: 10.12688/f1000research.13724.1 (PMC5909041; doi:10.12688/f1000research.13724.1)
Supplement: Peer review history

# Peer review: Referee response for version 1

**DOI:** 10.5256/f1000research.14908.r33175

**Published:** 2018-04-24

**Authors:** Svetlana V. Malysheva

**Affiliations:** 1 Russian Academy of Sciences , Moscow, Russian Federation

As the title implies the article describes the method of microsatellite loci isolation from the DNA of two sea anemone species, which are not the model objects. The goal of the study is to bring some information about 41 microsatellite loci and corresponding primers for PCR than can be used for different species of sea anemones, which genomes have not been sequenced yet. It is reported that 13 of 41 microsatellite loci were polymorphic for the two species but possibly will be suitable for population genetic studies of other sea anemones. Much attention is given to the otimization of PCR conditions and this procedure is described in detail. In my opinion, this article is interesting and can be useful for scientists who deal with sea anemones or other representatives of Anthozoa because it includes elaborate technique for microsatellite loci isolation as well as prepared loci for microsatellite analysis. The manuscript is certainly worthy for publication.

I have read this submission. I believe that I have an appropriate level of expertise to confirm that it is of an acceptable scientific standard.

# Peer review: Referee response for version 1

**DOI:** 10.5256/f1000research.14908.r33174

**Published:** 2018-04-18

**Authors:** Fabián H. Acuña

**Affiliations:** 1 Laboratory of Cnidarian Biology (LABIC), Faculty of Natural and Exact Sciences, Institute of Marine and Coastal Research (IIMyC), National Scientific and Technical Research Council (CONICET), National University of Mar del Plata (UNMDP), Mar del Plata, Argentina

This article is a concise study concerning with the identification of microsatellite loci in sea anemones *Aulactinia stella* and *Cribrinopsis albopunctata, *both belonging to family Actiniidae, from Avachinsky Bay of Kamchatka Peninsula. In the introduction the authors said "Sea anemones are known to live in clonal or partially clonal populations" but this is valid only for some (not all) sea anemone species and it is not possible to generalize. The metodology is detailed and all the source data are available to ensure full reproducibility. The authors mention that total DNA from pedal disc tissues of studied species was extracted, but they must justify why they choose this part and not other (column, oral disc or tentacles) of examined sea anemones for DNA extraction. The text is correctly written and obtained results are well exposed in the text and table 1. This article is a valuable contribution for sea anemone genetic population investigations and merit approval for indexing.

I have read this submission. I believe that I have an appropriate level of expertise to confirm that it is of an acceptable scientific standard.

# Peer review: Referee response for version 1

**DOI:** 10.5256/f1000research.14908.r31280

**Published:** 2018-03-23

**Authors:** Boris Levin

**Affiliations:** 1 Papanin Institute for Biology of Inland Waters, Russian Academy of Sciences, Yaroslavl, Russian Federation

MS ‘Identification of microsatellite loci in sea anemones *Aulactinia stella* and *Cribrinopsis albopunctata *(family Actiniidae)’ by E.S. Bocharova *et* *al.* is a methodical study related to the search and testing of polymorphic microsatellite loci in the sea anemones of above mentioned species of Actiniidae. Microsatellites are informative genetic markers for population genetic studies. Such approach is useful for obtaining information on many fundamental issues related to the reproductive strategy and spread of clonal organisms. Unlike well-studied commercial species, the biology of sea anemones is still remained unexplored in many aspects. Theory explaining the evolution of a larval stage and their long-distance dispersal ability requires genetic examination. The clonal nature of these organisms impedes application of mtDNA polymorphism and requires the search for other informative genetic markers such as microsatellites. The methods used in this study are modern and reproducible. Manuscript is well written and concise. Apparently, developed STR markers will be suitable in studies of other sea anemone species. I recommend the MS to be indexed.

I have read this submission. I believe that I have an appropriate level of expertise to confirm that it is of an acceptable scientific standard.
